# Effects of short-term hyperoxemia on cerebral autoregulation and tissue oxygenation in acute brain injured patients

**DOI:** 10.3389/fphys.2023.1113386

**Published:** 2023-02-08

**Authors:** Pietro Ciliberti, Danilo Cardim, Alberto Giardina, Matjaž Groznik, Lorenzo Ball, Martina Giovannini, Denise Battaglini, Erta Beqiri, Basil Matta, Peter Smielewski, Marek Czosnyka, Paolo Pelosi, Chiara Robba

**Affiliations:** ^1^ Department of Surgical Sciences and Integrated Diagnostics, University of Genoa, Genoa, Italy; ^2^ Department of Neurology, University of Texas Southwestern Medical Center, Dallas, TX, United States; ^3^ Institute for Exercise and Environmental Medicine, Texas Health Presbyterian Hospital, Dallas, TX, United States; ^4^ Traumatology Department of the University Clinical Center Ljubljana, Ljubljana, Slovenia; ^5^ Anesthesia and Intensive Care, San Martino Policlinico Hospital, IRCCS for Oncology and Neuroscience, Genoa, Italy; ^6^ Brain Physics Laboratory, Division of Neurosurgery, Department of Clinical Neurosciences, University of Cambridge, Cambridge, United Kingdom; ^7^ Neurocritical Care Unit, Addenbrooke’s Hospital, Cambridge University Hospitals NHS Foundation Trust, Cambridge, United Kingdom; ^8^ Institute of Electronic Systems, Warsaw University of Technology, Warsaw, Poland

**Keywords:** hyperoxygenation, cerebral autoregulation, brain injury, intracranial pressure, cerebral oxygenation

## Abstract

**Introduction:** Potential detrimental effects of hyperoxemia on outcomes have been reported in critically ill patients. Little evidence exists on the effects of hyperoxygenation and hyperoxemia on cerebral physiology. The primary aim of this study is to assess the effect of hyperoxygenation and hyperoxemia on cerebral autoregulation in acute brain injured patients. We further evaluated potential links between hyperoxemia, cerebral oxygenation and intracranial pressure (ICP).

**Methods:** This is a single center, observational, prospective study. Acute brain injured patients [traumatic brain injury (TBI), subarachnoid hemorrhage (SAH), intracranial hemorrhage (ICH)] undergoing multimodal brain monitoring through a software platform (ICM+) were included. Multimodal monitoring consisted of invasive ICP, arterial blood pressure (ABP) and near infrared spectrometry (NIRS). Derived parameters of ICP and ABP monitoring included the pressure reactivity index (PRx) to assess cerebral autoregulation. ICP, PRx, and NIRS-derived parameters (cerebral regional saturation of oxygen, changes in concentration of regional oxy- and deoxy-hemoglobin), were evaluated at baseline and after 10 min of hyperoxygenation with a fraction of inspired oxygen (FiO_2_) of 100% using repeated measures *t*-test or paired Wilcoxon signed-rank test. Continuous variables are reported as median (interquartile range).

**Results:** Twenty-five patients were included. The median age was 64.7 years (45.9–73.2), and 60% were male. Thirteen patients (52%) were admitted for TBI, 7 (28%) for SAH, and 5 (20%) patients for ICH. The median value of systemic oxygenation (partial pressure of oxygen-PaO_2_) significantly increased after FiO_2_ test, from 97 (90–101) mm Hg to 197 (189–202) mm Hg, *p* < 0.0001. After FiO_2_ test, no changes were observed in PRx values (from 0.21 (0.10–0.43) to 0.22 (0.15–0.36), *p* = 0.68), nor in ICP values (from 13.42 (9.12–17.34) mm Hg to 13.34 (8.85–17.56) mm Hg, *p* = 0.90). All NIRS-derived parameters reacted positively to hyperoxygenation as expected. Changes in systemic oxygenation and the arterial component of cerebral oxygenation were significantly correlated (respectively ΔPaO_2_ and ΔO_2_Hbi; r = 0.49 (95% CI = 0.17–0.80).

**Conclusion:** Short-term hyperoxygenation does not seem to critically affect cerebral autoregulation.

## Introduction

Acute brain injury is a major cause of mortality and severe disability (Maas et al., 2022). The outcome of these patients is dramatically affected by the quality of treatment in the first hours after the injury and by the prevention of secondary damage in the following hours (Maas et al., 2017; Steyerberg et al., 2019). Secondary brain injury is caused by several mechanisms, including hypotension, hypoxemia, altered cerebral autoregulation and intracranial hypertension (Volpi et al., 2018). More recently, not only the effect of hypoxemia, but also of hyperoxemia has been considered as possible cause of cerebral damage (Robba et al., 2022).

Oxygen is essential for aerobic respiration within mitochondria, but mitochondrial respiration also produces reactive oxygen species (ROS). Excessive administration of oxygen can consequently increase ROS formation, and induce activation of the inflammatory responses, which may cause further cerebral damage (Singer et al., 2021). Acute hyperoxemia can also cause vasoconstriction resulting in reduction of local blood flow, particularly in cerebral and coronary vessels, and possibly alter the vasomotor response of cerebral vessels (Rockswold et al., 2013). Current guidelines (Robba et al., 2020) suggest maintaining an arterial partial pressure of oxygen (PaO_2_) target of 80–120 mm·Hg in the acute phase of acute brain injury. In patients with traumatic brain injury (TBI), PaO_2_ values of 150–200 mm·Hg seem to be associated with better functional and cognitive outcome at 6 months, while PaO_2_ >200 mm·Hg was found to be independently associated with 6-months mortality (Alali et al., 2019). Furthermore, more recently, Rezoagli et al. (Rezoagli et al., 2022) demonstrated that in a large multicenter cohort of TBI patients (*n* = 1,084) the exposure to higher PaO_2_ and fraction of inspired oxygen (FiO_2_) in the first 7 days after ICU admission was independently associated with a higher 6-month mortality, independently of injury severity.

A specific threshold for oxygen neurotoxicity has not yet been identified, as well as the effect of hyperoxemia on cerebral autoregulation (CA) has never been evaluated thoroughly so far. In 2007, Nishimura et al. (2007) evaluated CA using transcranial Doppler in a cohort of healthy volunteers during hyperoxemia, suggesting that dynamic CA may remain unchanged, even with apparent changes in steady-state cerebral blood flow velocities.

At present, the most established method to assess cerebral autoregulation in patients with ICP monitoring in real-time is through the pressure reactivity index (PRx), which is a moving Pearson correlation between slow waves in intracranial pressure (ICP) and arterial blood pressure (ABP). Numerous retrospective studies in different patient populations demonstrated strong correlations between averaged PRx and outcome (Czosnyka et al., 1997; Sorrentino et al., 2012).

The primary aim of this study is to evaluate the short-term effects of hyperoxemia (obtained through FiO_2_ = 100%) on cerebral autoregulation assessed via PRx. Secondary outcomes include the assessment of the effect of hyperoxemia on cerebral oxygenation and ICP, and the correlation between changes in cerebral autoregulation and systemic as well as cerebral oxygenation parameters.

## Methods

Single center, prospective observational study, conducted from first of January 2021 to first of September 2022, at Policlinico San Martino Hospital, IRCCS for Oncology and Neuroscience, Genova, Italy. The study was approved by the local ethics review board (Comitato Etico Regione Liguria, protocol n. CER Liguria: 23/2020). Approval for the study was obtained according to local regulations. This study was performed according to the “Strengthening the Reporting of Observational Studies in Epidemiology (STROBE)” statement guidelines for observational cohort studies (von Elm et al., 2014) (Additional file 1: ESM Supplementary Table S1). Inclusion criteria were patients above 18 years of age, admitted to the intensive care unit (ICU), following acute brain injury [ABI, defined as TBI, or cerebrovascular disease, such as subarachnoid hemorrhage (SAH) or spontaneous intracranial hemorrhage (ICH)], intubated and mechanically ventilated undergoing multimodal neuromonitoring, assigned to beds equipped with of ICM + software (Smielewski et al., 2005) (according to clinical severity and based on bed availability) and receiving pre-oxygenation with 100% FiO_2_ for clinical reasons at bedside for optimization of respiratory management. Our unit is a mixed general and neuro-ICU, composed of 28 level 3 beds, of which 12 are equipped with ICM + software. Patients without multimodal monitoring, not mechanically ventilated or not receiving pre-oxygenation with 100% FiO_2,_ or those patients whose next of kins refused to agree about inclusion in the study were excluded.

### Data collection

#### Demographics

Demographical (age, gender, body mass index), clinical data (pre-injury comorbidities such as respiratory, cardiovascular, liver and kidney disease, cancer, diabetes mellitus and hypertension), reason for ICU admission (i.e., TBI, SAH, ICH), neurological status at admission as for Glasgow coma scale (GCS), pupils characteristics (reactivity, iso or anisocoria), type of ICP monitoring (intraparenchymal or external ventricular drain), ICU complications (i.e., acute distress respiratory syndrome, ventilator-associated pneumonia, acute kidney injury, sepsis, vasospasm) and patients’ outcome, such as ICU length of stay, mortality and neurological status (Glasgow Outcome Score) at ICU discharge were collected from electronic medical records.

### General ICU management

In the ICU, patients were sedated with Propofol/Midazolam and Fentanest and were mechanically ventilated using an endotracheal tube. Tidal volume was targeted to 6–8 mL per kg of predicted body weight (PBW). Higher values of tidal volume were allowed only if the airway driving pressure was maintained below 15 cm H_2_O. Respiratory rate was adjusted to aim to normocapnia. The decision to perform an FiO_2_ trial was based on clinician’s evaluation when optimization of mechanical ventilation was required occurring within 24 h of hospital admission, in order to obtain an estimation of the presence of shunt or low ventilation/perfusion ratio and as part of the evaluation of the respiratory function performed in our ICU (Aboab et al., 2006; Ball et al., 2021). Data on neuromonitoring were obtained at baseline (T0) with FiO_2_ set as for clinical needs, and after the test (T1) which consisted of applying FiO_2_ set at 100% (during 10 min for stabilization), as previously described (Cressoni et al., 2017). Arterial blood gases, such as partial pressure of oxygen (PaO_2_) and of carbon dioxide (PaCO_2_) before and after FiO_2_ test were also collected.

### Multimodal neuromonitoring and management

Invasive ICP monitoring was inserted for clinical reasons according to our local policies following the latest Brain Trauma Foundation Guidelines (Carney et al., 2017). Patients’ clinical management of intracranial hypertension was performed according to the Seattle algorithm (Chesnut et al., 2020). ICP was monitored continuously with a transducer into the parenchymal space or through an external cerebral fluid shunt. ABP was monitored in the radial or femoral artery zeroed at the level of the right atrium (Baxter Healthcare, CA, United States; Sidcup, United Kingdom). In patients with head elevation, no corrections were made for hydrostatic pressure differences. For the assessment of cerebral oxygenation, we used non-invasive continuous regional cerebral oxygen saturation using the Root^®^ with O_3_^®^ regional oximetry device (Masimo, CA, United States), with a bilateral sensor applied in the frontotemporal region. Final values of rSO_2_ and its components at T0 and T1 were calculated as the mean between single instant measurements obtained from the right and left frontotemporal sensors. Different parameters of cerebral oxygenation can be obtained from this monitor: a) rSO_2_, which represents the regional cerebral oxygen saturation, and is derived as the ratio of the concentration of oxyhemoglobin (O_2_Hb) and total hemoglobin (cHb = O_2_Hb + HHb, where HHb is deoxyhemoglobin); b) ΔO_2_Hbi, which is an index associated with changes of concentration of oxyhemoglobin, thus representing predominantly changes in the arterial component of regional oxygen saturation; c) ΔHHbi, an index reflecting changes in concentration of deoxyhemoglobin, approximately representing changes in the venous component of the oxygen saturation; d) ΔcHbi, an index representing the sum of ΔO_2_Hbi and ΔHHbi components (Robba et al., 2021a). All continuous physiological data were collected simultaneously and analyzed using ICM+ (Cambridge Enterprise, Cambridge, United Kingdom, https://icmplus.neurosurg.cam.ac.uk) (Smielewski et al., 2005), a clinical research software which can provide real-time analysis of multimodal monitoring modalities at the patient’s bedside. Data collected with ICM+ were sampled at 100 Hz. Artifacts were visually inspected and manually removed from the data time series using artifact removal tools on ICM+. Cerebral autoregulation assessed through PRx was calculated over a 5-min moving window as the Pearson correlation of 30 consecutive 10-s average values of ABP and ICP as previously described (Donnelly et al., 2015). A preserved autoregulation was defined as values of PRx below 0.3 whereas higher values are defined as altered CA (Donnelly et al., 2018; Sorrentino et al., 2012). PRx was calculated from T0 (averaged value of a 10-min period before FiO_2_ test) and T1 (averaged value of a 10-min period immediately after FiO_2_ test) periods.

### Statistical analysis

The Shapiro-Wilk test was used to test the normality of the distribution of the variables. Continuous variables are reported as median and interquartile range (IQR = 25th–75th percentiles). Comparisons between different variables at T0 and T1 were made by repeated measures (paired) *t*-test for normally distributed variables, while non-normally distributed variables were compared by paired Wilcoxon signed-rank test. Graphical representations of these comparisons are presented as boxplots. Dependent variables were expressed as a change from baseline (T0) in absolute terms (Δ change = T1-T0). The correlations coefficients (95% confidence interval (CI)) between systemic and the different neuromonitoring variables were verified using Pearson’s or Spearman’s method, for parametric and non-parametric variables, respectively. All statistical analyses were performed using RStudio software (version 4.1.1). A *p*-value <0.05 was considered statistically significant.

## Results

During the study period, a total of 110 patients with ABI were admitted to our ICU and were considered for inclusion. Fifty-two patients were excluded as they did not undergo multimodal neuromonitoring while 33 patients were not allocated to beds equipped with multimodal monitoring capability where ICM+ software was installed. A final number of 25 patients were included in the analysis. The characteristics of the patients are presented in Table 1. The median age was 64.7 years (45.9–73.2), and 60% were male. Thirteen patients (52%) were admitted for TBI, 7 (28%) for SAH, and 5 (20%) patients for ICH. Hypertension was the most common preinjury comorbidity (6 patients, 24%), and five patients (20%) were smokers. At ICU discharge, five patients had died (20%), and median GOS was 3 (1.8–4.0). According to the Seattle algorithm for patient management (Chesnut et al., 2020), of the 25 patients included none of them was in tier 3, 13 were in tier 1, and 12 were in tier 2.

**TABLE 1:** Demographics, general characteristics, intensive care unit complications.

General characteristics	
Age, years, median [IQR]	64.7 [45.9; 73.2]
BMI, kg/m^2^, median [IQR]	25.0 [23.4; 26.1]
Gender, male/female, n (%)	15/10 (60/40)
Comorbidities	
None, n (%)	6 (24)
≥2 comorbidities, n (%)	9 (36)
Smoke habits, n (%)	5 (20)
Hypertension, n (%)	6 (24)
Diabetes, n (%)	1 (4)
Other endocrine/ metabolic disease, n (%)	3 (12)
Alcohol and/or drugs abuse, n (%)	1 (4)
Kidney injury, n (%)	1 (4)
Liver injury, n (%)	1 (4)
Depression and/or anxiety, n (%)	4 (4)
Cardiovascular disease, n (%)	1 (4)
Neurological disease, n (%)	3 (12)
Neurological disease and severity	
Out-of-hospital GCS, points, median [IQR]	6 [3; 11.5]
Anisocoria, n (%)	8 (32)
Type of brain injury, n (%)	
SAH	7 (28)
TBI	13 (52)
ICH	5 (20)
Type of invasive monitoring, n (%)	
EVD,	9 (36)
Intraparenchymal bold,	16 (64)
TBI, Marshall classification, n (%)	
I	0 (0)
II	0 (0)
III	5 (38.4)
IV	4 (30.8)
V	4 (30.8)
VI	0 (0)
SAH, Fisher classification, n (%)	
I	0 (0)
II	0 (0)
III	0 (0)
IV	7 (100)
ICU outcomes and complications	
GOSE, points, median [IQR]	3.0 [1.8; 4.0]
ICU complications, n (%)	
≥2 complications	8 (32)
None	7 (28)
Septicemia	13 (52)
VAP	8 (32)
Acute kidney injury	1 (4)
Meningitis	1 (4)
Epileptic crisis	2 (8)
Hydrocephalus	1 (4)
Vasospasm after SAH	1 (4)
ICU-LOS, days, median [IQR]	32.0 [20.0; 52.5]
MV duration, days, median [IQR}	16.0 [9.5; 26.5]
Mortality, n (%)	5.0 (20)

BMI, body mass index; GCS, Glasgow coma scale; IQR, interquartile range; SAH, subarachnoid hemorrhage; TBI, traumatic brain injury; ICH, intracranial haemorrhage; EVD, external ventricular drain; GOSE, extended Glasgow outcome scale; ICU, intensive care unit; VAP, ventilator associated pneumonia; LOS, length of stay; MV, mechanical ventilation.

### Effect of FiO_2_ test on cerebral and systemic factors

**FIGURE 1:**
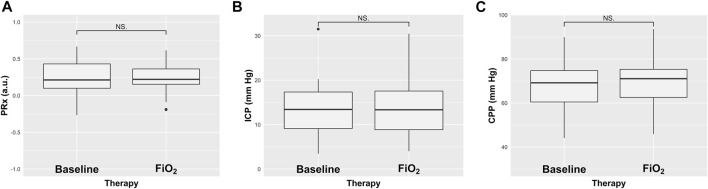
Boxplots representing the effect of increased fraction of inspired oxygen (FiO_2_) on cerebral autoregulation measured with pressure reactivity test (PRx) **(A)**, intracranial pressure (ICP) **(B)**, and cerebral perfusion pressure (CPP) **(C)** from baseline. Values are presented as median and interquartile range.

**FIGURE 2:**
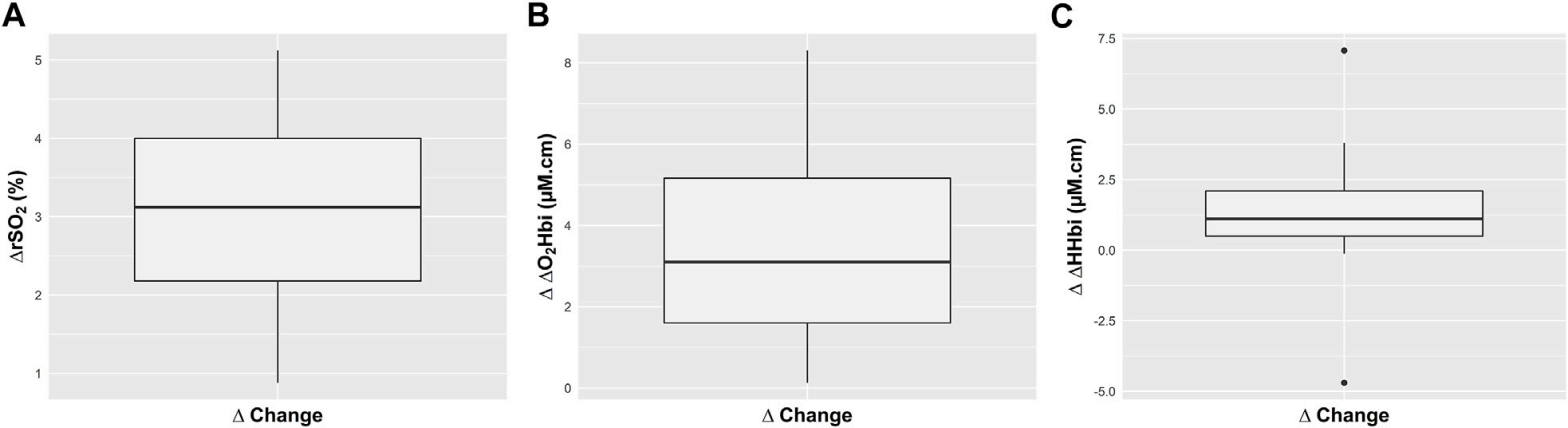
Boxplots representing the effect of fraction of increased inspired oxygen (FiO_2_) on absolute changes (∆) in regional cerebral oxygen saturation (rSO_2_) **(A)**, and in the arterial (ΔO_2_Hbi) **(B)** and venous (ΔHHbi) **(C)** component of cerebral oxygenation. Values are presented as median and interquartile range.

The median value of PaO_2_ significantly increased after FiO_2_ test, from 97 (90–101) mm·Hg to 197 (189–202) mm·Hg, *p* < 0.0001 (Table 2). Similarly, SpO_2_ increased after FiO_2_ test, from 96 (95–97) % to 100 (100–100) %, *p* < 0.0001 (paired Wilcoxon test), but PaCO_2_ did not significantly change (42 (39–44) mm·Hg at T0 and 42 (38–45) mm·Hg at T1, *p* = 1) (ESM, Supplementary Figure S1). After FiO_2_ test, no statistically significant changes were observed in PRx values (from 0.21 (0.10–0.43) to 0.22 (0.15–0.36), *p* = 0.68), as well as on ICP (from 13.42 (9.12–17.34) mm·Hg to 13.34 (8.85–17.56) mmHg, *p* = 0.99), and CPP values (from 69.20 (60.47–74.72) mm Hg to 71.08 (62.56–75.34) mm Hg, *p* = 0.76) (Table 2; Figure 1). On an individual basis, nine patients had PRx >0.3 at T0 and T1, consisting of the same individuals at both timepoints. All NIRS parameters increased significantly after FiO_2_ test (Δ absolute changes). rSO_2_ increased by 3.12 (2.18–4.00) %, *p* < 0.0001; ΔO_2_Hbi by 3.10 (1.60–5.16) μM.cm, *p* < 0.0001; and ΔHHbi by 1.11 (0.49–2.10) μM.cm, *p* < 0.0001 (paired Wilcoxon test), respectively (Figure 2).

**TABLE 2:** Systemic and neuromonitoring data before and after the FiO_2_ test. Data are presented as median and interquartile range.

	FiO_2_ 50% (T0)	FiO_2_ 100% (T1)	Δ change	p-value
ICP	13.42 (9.12-17.34)	13.34 (8.85-17.56)	-0.10 (-0.90-0.44)	0.14
CPP	69.20 (60.47-74.72)	71.08 (62.56-75.34)	1.14 (0.55-2.12)	0.16
MAP	82.66 (76.00-87.56)	83.43 (75.00-88.74)	1.01 (0.38-2.03)	0.26
PRx	0.21 (0.10-0.43)	0.22 (0.15-0.36)	0.02 (-0.02-0.11)	0.30
rSO_2_	61 (57-65)	65 (60-70)	3.12 (2.18-4.00)	**<0.0001**
ΔO_2_Hbi	2.70 (1.60-4.70)	6.50 (4.30-8.20)	3.10 (1.60-5.16)	**<0.0001**
ΔHHbi	2.21 (1.62-3.02)	3.40 (2.80-5.40)	1.11 (0.49-2.10)	**<0.0001**
SPO_2_	96 (95-97)	100 (100-100)	4 (3.00-4.17)	**<0.0001**
PaO_2_	97 (90-101)	197 (189-202)	101 (95-107)	**<0.0001**
PaCO_2_	42 (39-44)	42 (38-45)	0 (-1-1)	1

Baseline (T0) and post-FiO_2_ test (T1) values of the different parameters. ICP: intracranial pressure (mm Hg); CPP: cerebral perfusion pressure (mm Hg); MAP: mean arterial blood pressure (mm Hg); PRx: pressure reactivity index; rSO_2_: regional tissue oxygen saturation (%); ∆O_2_Hbi: index representing the change in the oxyhemoglobin of the regional tissue oxygen saturation (μM.cm); ∆HHbi: index representing the change in the deoxyhemoglobin of the regional tissue oxygen saturation (μM.cm); SpO_2_: systemic oxygen saturation (%); PaO_2_: partial pressure of O_2_ (mm Hg); PaCO_2_: partial pressure of CO_2_ (mm Hg).

### Correlation between changes in systemic and neuromonitoring parameters

**FIGURE 3:**
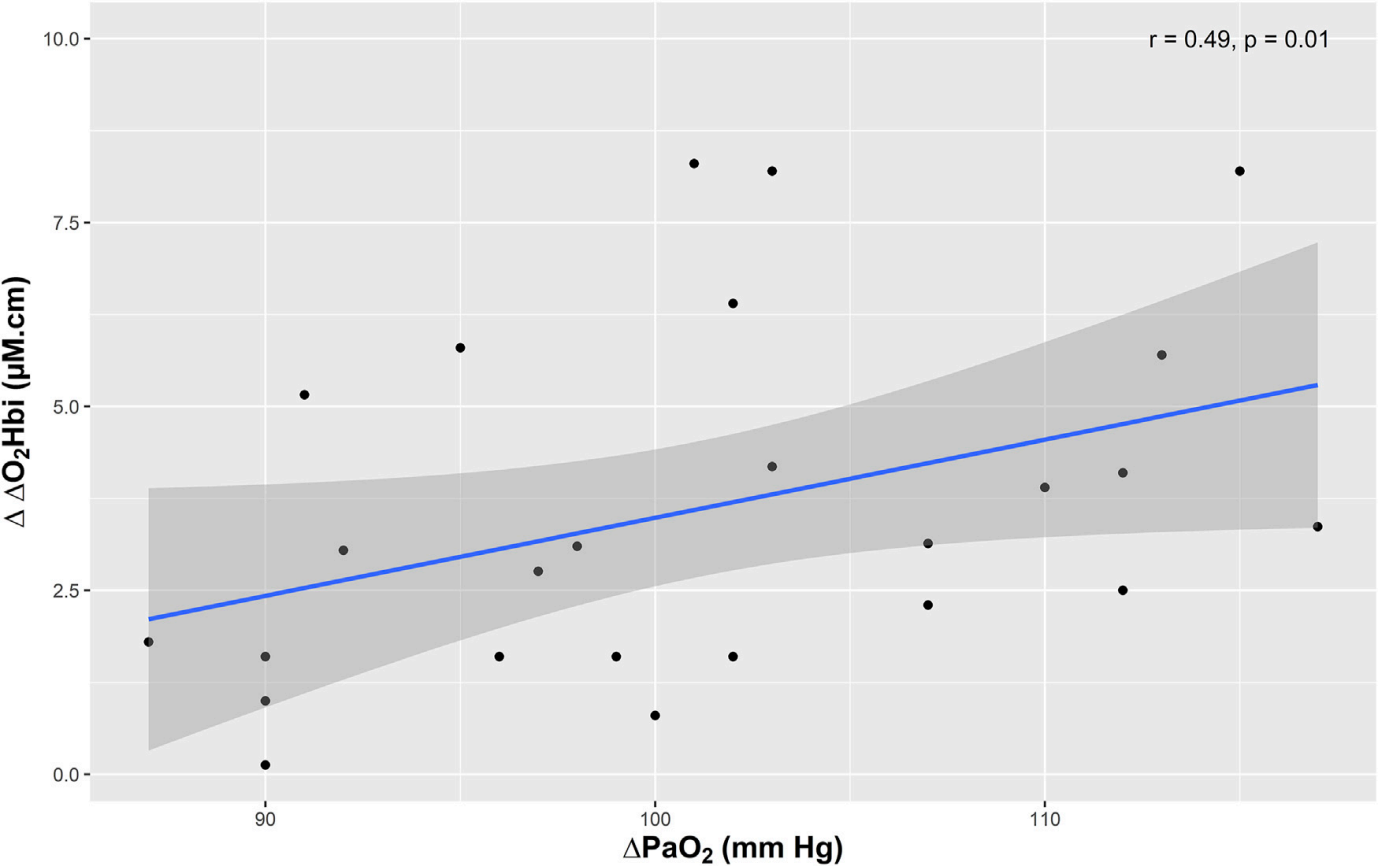
Scatter plots showing the correlation (r) between changes in the arterial component of cerebral oxygenation (ΔO_2_Hbi) and partial pressure of oxygen (PaO_2_).

Absolute changes in systemic oxygenation and concentration of oxygenated hemoglobin were significantly correlated [ΔPaO_2_ and Δ ΔO_2_Hbi, r = 0.49 (95% CI = 0.17–0.80), *p* = 0.01 (Spearman’s rank correlation coefficient)] (Figure 3). No other significant correlation was found between the changes of PRx, ICP and other multimodal monitoring parameters with systemic parameters (Table 3).

**TABLE 3:** Correlation between changes(Δ) in systemic and neuromonitoring parameters.

	ΔICP	ΔPRx	ΔSpO_2_	ΔrSO_2_	ΔO_2_Hbi	ΔHHbi	ΔPaO_2_
ΔICP							
r	1.00	0.30	-0.18	-0.01*	0.15	-0.23	0.22*
p-value	-	0.15	0.39	0.94	0.49	0.27	0.30
ΔPRx							
r	0.30	1.00	0.14	-0.35	0.14	-0.12	0.04
p-value	0.15	-	0.50	0.09	0.52	0.57	0.84
ΔSpO_2_							
r	-0.18	0.14	1.00	-0.02	-0.03	-0.16	0.39
p-value	0.39	0.50	-	0.91	0.88	0.44	0.05
ΔrSO_2_							
r	-0.01*	-0.35	-0.02	1.00	-0.07	-0.04	0.01*
p-value	0.94	0.09	0.91	-	0.76	0.83	0.95
ΔO_2_Hbi							
r	0.15	0.14	-0.03	-0.07	1.00	0.23	**0.49**
p-value	0.49	0.52	0.88	0.76	-	0.28	**0.01**
ΔHHbi							
r	-0.23	-0.12	-0.16	-0.04	0.23	1.00	0.07
p-value	0.27	0.57	0.44	0.83	0.28	-	0.73
ΔPaO_2_							
r	0.22*	0.04	0.39	0.01*	**0.49**	-0.07	1.00
p-value	0.30	0.84	0.05	0.95	**0.01**	0.73	-

**r:** correlation coefficient. * represents Pearson correlation coefficients; the remaining values represent Spearman correlation coefficients.

ICP: intracranial pressure; CPP: cerebral perfusion pressure; PRx: pressure reactivity index; rSO_2_: regional tissue oxygen saturation; ∆O_2_Hbi: index representing the change in the oxyhemoglobin of the regional tissue oxygen saturation; ∆HHbi: index representing the change in the deoxyhemoglobin of the regional tissue oxygen saturation; SpO_2_: systemic oxygen saturation; PaO_2_: partial pressure of O_2_.

## Discussion

In patients with ABI and mechanically ventilated, we found that the increase in systemic PaO_2_ at FiO_2_ 100% oxygen for 10 min does not affect cerebral autoregulation. The use of specific indices derived from NIRS allowed for hypothesizing on the arterial and venous components of cerebral oxygenation and thus the pathophysiological interplay between systemic and cerebral oxygenations and CA.

To our knowledge, this is the first physiological study assessing the effect of hyperoxemia on CA using continuous monitoring of cerebrovascular pressure reactivity. Previous studies have questioned the effect of hyperoxemia on cerebral dynamics, but without focusing on the importance of cerebral autoregulation. This is clinically relevant, as recent evidence suggests that hyperoxemia can be detrimental to ABI patients and has been associated with poor neurological outcome and independently associated with higher in-hospital fatality (Rincon et al., 2014; Hirunpattarasilp et al., 2022). Cerebral autoregulation is a compensatory mechanism of the brain that allows maintenance of constant cerebral blood flow (CBF) regardless of changes in perfusion pressure (Claassen et al., 2021). The blood pressure range within which cerebral autoregulation is preserved is wide in healthy individuals. In patients with acute brain injuries, this range narrows and the risk of secondary damage increases (Donnelly et al., 2015). Impaired CA has been shown to be associated with worse outcome, highlighting the importance of its assessment (Rincon et al., 2014; Hirunpattarasilp et al., 2022).

PRx is an index of cerebrovascular reactivity which assesses the response of ICP to spontaneous vasogenic changes in ABP (Czosnyka et al., 1997). A positive PRx means a positive gradient of the regression line between the slow oscillations of ABP and ICP, which are associated with passive behavior of non-reactive cerebral arterioles. In this case, an increase in ABP would cause the arterioles to passively dilate, thus the cerebral blood volume would increase, leading to an increase in ICP. Instead, a negative value of PRx reflects reactive cerebral arterioles which actively counteract a change in CPP to maintain CBF stable (Sorrentino et al., 2012). PRx is described as a surrogate index of global cerebral autoregulation. Although other tools are currently available for CA assessment, PRx is considered the most reliable method in neurocritical care patients requiring ICP monitoring. It is the only index validated against the gold standard static methods of direct cerebral perfusion assessment (Steiner et al., 2003; Brady et al., 2007; Depreitere et al., 2021).

Hypoperfusion and hypoxemia are common mechanisms of secondary damage after ABI (Volpi et al., 2018); however, very little is known about the effect of hyperoxemia (Raj et al., 2013; Robba et al., 2021b). Breathing 100% oxygen in normal subject results in vasoconstriction of cerebral blood vessels, which decreases CBF and increases brain tissue oxygenation pressure. However, in TBI, the vascular and tissue responses to hyperoxemia may work differently and may potentially lead to an alteration of the vasomotor response and therefore of autoregulation (Johnston et al., 2003). As discussed by Rezoagli et al. (2022) in their study with TBI patients, increasing PaO_2_ does not necessarily increase the delivery of oxygen in the injured brain parenchyma. This is related to the fact that oxygen flux is complexly governed by a combination of diffusion and consumption rather than simply tissue difusion (Ercole, 2022). These considerations indicate that the use of hyperoxia, even in the presence of low PbtO_2_ may be harmful rather than beneficial, given hyperoxia was independently associated with a higher 6-month mortality rate (Ercole, 2022; Rezoagli et al., 2022). At present, findings regarding which hyperoxemia target should be used in ABI patients are inconclusive, and current guidelines only suggest targeting PaO_2_ values to a range of 80–120 mm·Hg^8^.

We found no significant change in PRx values after the FiO_2_ test, which suggests that cerebral autoregulation was, at least, not critically affected by the challenge. This analysis considered the nine patients that had unchanged impaired CA status (PRx >0.3) at both T0 and T1. Moreover, increased systemic PaO_2_ promoted significant changes in all NIRS parameters, ΔO_2_Hbi, ΔHHbi and rSO_2_, suggesting a probable increase in both hemoglobin O_2_ saturation and cerebral blood flow, thus decreasing O_2_ extraction fraction. On the other hand, the observed change in PaO_2_ in our cohort after hyperoxia (ΔPaO_2_) was only directly correlated with the absolute changes in the arterial component of cerebral oxygenation (as indicated by Δ ΔO_2_Hbi). This suggests a potential modulation of the arterial component of cerebral oxygenation by increased systemic oxygenation.

A recently published study investigating the effects of hyperoxia on cerebral blood flow velocity and regional oxygen saturation in patients with focal severe TBI found that an increase in rSO_2_ from normal levels is observed after hyperoxemia only when CA is impaired (Sahoo et al., 2017). This finding suggests that arterial hyperoxia does not lead to an increase in cerebral oxygenation when autoregulation is intact. This previous study (Sahoo et al., 2017) had a considerably higher absolute change in rSO_2_ after hyperoxia in individuals presenting impaired CA compared to our findings presented here, approximately 11% vs. 3%, respectively. In our case, the substantially smaller change might represent a larger individual variability within patients rather than a true increase in rSO_2_ caused by impaired CA, or due to the possibility that CA was only partially preserved before and after hyperoxia in our cohort.

PRx, as a means to assess CA globally, reflects largely the myogenic component (pressure reactivity), and to a lesser extent the metabolic component (i.e., cerebrovascular response to O_2_ and CO_2_ changes) of cerebral blood flow regulation. Cerebrovascular reactivity to oxygen, for instance, is hampered when cerebral autoregulation is impaired, so that rSO_2_ increases passively with increasing PaO_2_. The physiological cerebrovascular response to significant hyperoxia is vasoconstriction (which promotes decrease in cerebral blood volume), while when oxygen reactivity is impaired hyperoxia does not change the vascular resistance (Sahoo et al., 2017). In our cohort, we observed an increase in cerebral blood volume, as indicated by the sum of positive changes in ΔO_2_Hbi and ΔHHbi (i.e., ΔcHbi). Despite a major increase in PaO_2_ (from 97 to 197 mm·Hg) after FiO_2_ test, we did not observe a considerable change in rSO_2_, nor was this correlated to changes in PaO_2_. This likely suggests that in our patient cohort CA was at least partially impaired, especially the metabolic component of CA which cannot be fully explained by PRx. Another important remark to consider in the interpretation of our findings is that CA was measured in the non-injured brain tissue, and while total brain PaO_2_ may be elevated, this increase may not be present in the injured tissue (Ercole, 2022). In this sense, the measure of CA is not a *de facto* measure of generalized brain health, particularly in regional settings of ABI, where ICP is measured outside of the injured site.

Several limitations of the present study should be addressed. First, the single center design and the small sample cohort limit the generalization of our results. Secondly, due to the lack of a control group, the impact of hyperoxemia on mortality and neurological outcome was not assessed. Thirdly, the FiO_2_ test was of short duration as for our clinical practice; a more prolonged test could help to better elucidate the long-term impact of hyperoxemia on brain oxygenation in patients with ABI. Further prospective confirmatory studies are necessary to identify a higher hyperoxia threshold which can potentially affect cerebral dynamics and outcome (Grensemann et al., 2022) and to properly define the optimal target of oxygen delivery in acute neurocritical care patient settings and its effects on cerebral dynamics and clinical outcomes. Furthermore, known limitations of NIRS, such as the potential influence of extracranial contamination (particularly in HHb and O_2_Hb signals), the nature of HHb and HbO_2_ dependent on the unknown scattering coefficient likely differing on an individual basis, and the unknown contribution of venous and arterial components to the measured signals, particularly the O_2_Hb, are potential confounders in our study.

## Conclusion

In mechanically ventilated neurocritical care patients with ABI, short-term hyperoxemia does not seem to critically affect cerebral autoregulation.

## Data Availability

The raw data supporting the conclusions of this article will be made available by the authors, without undue reservation.
