# Supplementary figures and images for: Effects of short-term hyperoxemia on cerebral autoregulation and tissue oxygenation in acute brain injured patients
Source: Front Physiol. 2023 Feb 8;14:1113386. doi: 10.3389/fphys.2023.1113386 (PMC9944047; doi:10.3389/fphys.2023.1113386)

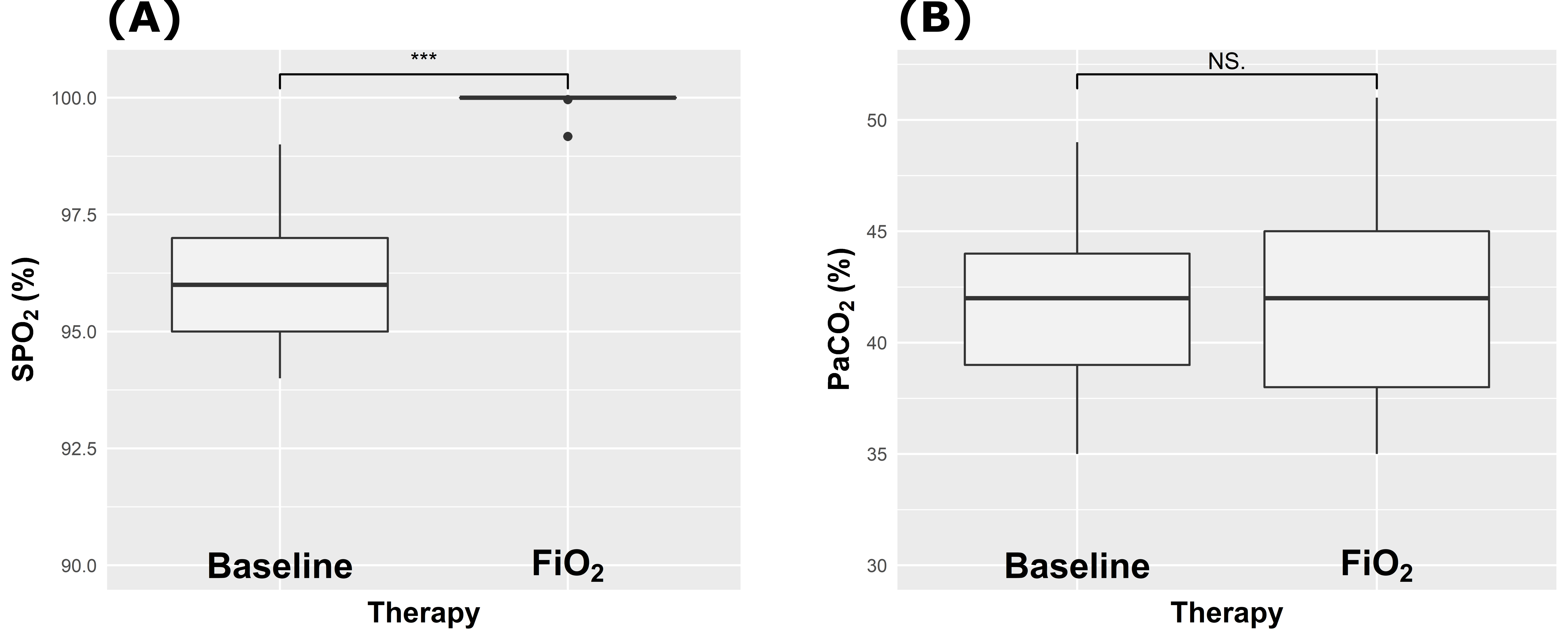

Supplement: Supplementary file 2 [file Image1.TIFF]
